# Alpha-Herpesvirus Thymidine Kinase Genes Mediate Viral Virulence and Are Potential Therapeutic Targets

**DOI:** 10.3389/fmicb.2019.00941

**Published:** 2019-05-08

**Authors:** Ying Xie, Liping Wu, Mingshu Wang, Anchun Cheng, Qiao Yang, Ying Wu, Renyong Jia, Dekang Zhu, XinXin Zhao, Shun Chen, Mafeng Liu, Shaqiu Zhang, Yin Wang, Zhiwen Xu, Zhengli Chen, Ling Zhu, Qihui Luo, Yunya Liu, Yanling Yu, Ling Zhang, Xiaoyue Chen

**Affiliations:** ^1^ Institute of Preventive Veterinary Medicine, Sichuan Agricultural University, Chengdu, China; ^2^ Key Laboratory of Animal Disease and Human Health of Sichuan Province, Sichuan Agricultural University, Chengdu, China; ^3^ Avian Disease Research Center, College of Veterinary Medicine, Sichuan Agricultural University, Chengdu, China

**Keywords:** α-Herpesvirus, thymidine kinase genes, thymidine kinases, viral virulence, latent infection, antiviral treatments, antitumor gene therapy, reporter genes

## Abstract

Alpha-herpesvirus thymidine kinase (TK) genes are virulence-related genes and are nonessential for viral replication; they are often preferred target genes for the construction of gene-deleted attenuated vaccines and genetically engineered vectors for inserting and expressing foreign genes. The enzymes encoded by TK genes are key kinases in the nucleoside salvage pathway and have significant substrate diversity, especially the herpes simplex virus 1 (HSV-1) TK enzyme, which phosphorylates four nucleosides and various nucleoside analogues. Hence, the HSV-1 TK gene is exploited for the treatment of viral infections, as a suicide gene in antitumor therapy, and even for the regulation of stem cell transplantation and treatment of parasitic infection. This review introduces the effects of α-herpesvirus TK genes on viral virulence and infection in the host and classifies and summarizes the current main application domains and potential uses of these genes. In particular, mechanisms of action, clinical limitations, and antiviral and antitumor therapy development strategies are discussed.

## Introduction

**Figure 1:**
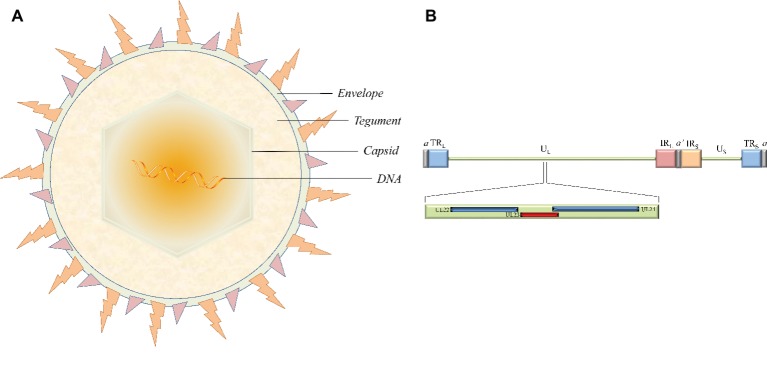
The structure of virion and the location of the thymidine kinase gene (UL23) in the HSV-1 genome. **(A)** The virion is mainly composed of four morphologically distinct structures. **(B)** HSV-1 genomes contain U_L_ and U_S_ regions that are each flanked by terminal and internal inverted repeats (TR_L_/IR_L_ and IR_S_/TR_S_). The UL23 gene is located between UL22 and UL24 in the U_L_ regions in the same orientation as the UL22 gene and the opposite orientation from the UL24 gene.

Herpesviruses are large double-stranded DNA (dsDNA) viruses with a size varying from 120 to as large as 260 nm. The virion is mainly composed of four morphologically distinct structures: a core containing the linear dsDNA, a highly stable icosahedral capsid made of 162 capsomeres, the largely unstructured protein tegument, and the outer lipid layer envelope (Figure 1A; Yuan et al., 2005; Guo et al., 2009b; Wu et al., 2012a,b). The latest report of the International Committee on Taxonomy of Viruses (ICTV) showed that the herpesvirus family is divided into three subfamilies, the *alphaherpesvirinae*, *betaherpesvirinae*, and *gammaherpesvirinae* (Adams et al., 2013). The *alphaherpesvirinae* subfamily mainly includes herpes simplex virus 1 and 2 (HSV-1 and -2), varicella-zoster virus (VZV), Marek’s disease virus (MDV), pseudorabies virus (PRV), and duck enteritis virus (DEV). Human and murine cytomegalovirus (HCMV and MCMV, respectively) and human herpes virus 6A, 6B, and 7 (HHV-6A, HHV-6B, and HHV-7, respectively) belong to the *betaherpesvirinae* subfamily. Epstein-Barr virus (EBV) and human herpes virus 8 (HHV-8) are well-known representatives of the *gammaherpesvirinae* subfamily (Qi et al., 2009; Guo et al., 2009a; Yang et al., 2010; Xiang et al., 2012; Liu et al., 2017; You et al., 2017, 2018; Zhao et al., 2018). These different virus species can induce acute and sometimes chronic contagious infections in their specific natural hosts like humans. In addition, the typical epidemiological feature of α-herpesviruses is the ability to establish latent infection, where the virus is carried by survivors for a long time and efficiently reactivates under certain circumstances. Periodic reactivation or intermittent cyclic activity of the latent virus results in recurrent disease that is usually mild but can be fatal in immunocompromised patients (Sili et al., 2014; Ramakrishna et al., 2015).

Thymidine kinase (TK, EC2.7.1.21), associated with nucleoside salvage pathway, exists widely in bacterial, eukaryotic, and prokaryotic cells (Black and Hruby, 1991; Ge et al., 2008; Han et al., 2008; Wen et al., 2010; Timm et al., 2015; Leija et al., 2016). Cellular TKs, including cytosolic TK1 and mitochondrial TK2, are the key enzymes that catalyze the transfer of the γ-phosphate of ATP to 2′-deoxythymidine (dT) in the nucleoside salvage pathway, forming thymidine monophosphate (dTMP) in the presence of magnesium ions (Mg^2+^). The vast majority of herpesviruses and some DNA viruses, such as vaccinia virus (VACV) (Deng et al., 2017), African swine fever virus (ASFV) (Sanford et al., 2016), and *Rana grylio* virus (RGV) (Zhao et al., 2009), also have a specific gene encoding a viral TK. For α-herpesviruses, but in particular for HSV-1, the expression of the viral TK is an important factor influencing virus characterization in acute and latent infection (Tenser et al., 1979; Huang et al., 2017). With broader substrate specificity than other viral and cellular TKs, HSV-1 TK can phosphorylate not only dT and deoxycytidine (dC) but also a series of nucleoside analogues such as acyclovir (ACV), which are recognized and activated as HSV-1 TK substrates (Deville-Bonne et al., 2010). Thus, TKs are crucial targets in antiherpesvirus treatments and potential therapeutic targets in antitumor gene therapy strategies (Sangro et al., 2010; James and Prichard, 2014).

In this review, the roles of the α-herpesvirus TK genes in viral virulence and latent infection, as well as the mechanism and development of antiviral drugs and antitumor gene therapies that target α-herpesvirus TK activity, are summarized. We also provide insight into some potential uses according to TK gene characteristics.

## Properties of α-Herpesviruses Thymidine Kinase Genes

The HSV-1 TK gene (NC_001806.2), also known as the UL23 gene, is located in the order of UL22-UL23-UL24 gene between the complementary sequences of the UL24 gene and the UL22 gene in the genome in the same orientation as the UL22 gene and with the opposite orientation from the UL24 gene (Figure 1B). The position of the TK gene sequence is similar in α-herpesviruses from different sources, but these other TK genes have low sequence homology with the HSV-1 TK gene. For example, the feline herpesvirus (FHV) TK gene and HSV-1 TK gene are only 31 and 35% homologous with the canine herpesvirus (CHV) TK gene, respectively (Solaroli et al., 2006). Surprisingly, betaherpesvirinae such as HCMV and MCMV lack the gene coding viral TK (Chee et al., 1990; Rawlinson et al., 1996). However, there are exceptions to this trend; endotheliotropic elephant herpesvirus 1 (EIHV-1) is the first reported β-herpesvirus TK gene, probably related to differences in viral evolution (Ehlers et al., 2006).

The HSV-1 TK gene expression is strictly regulated following timing principles and cascading effects. The three general kinetic classes of genes are designated the immediate early (IE or α) genes, the early (E or β) genes, and the late (L or γ) genes (Honess and Roizman, 1974). These different types of genes regulate each other’s transcription. Five IE proteins (ICP0, ICP4, ICP22, ICP27, and US1.5) activate the transcription of E genes, at least in several types of cells, and the transcription of L genes depends on the activation of E proteins. On the other hand, inhibition of E gene transcription slows the synthesis rate of IE proteins, and transcriptional activation of L genes can terminate the expression of E proteins at a later stage (Honess and Roizman, 1975; Kukhanova et al., 2014). As a member of the herpesvirus E gene family, the TK gene can be expressed at a basal level in heterologous systems such as mouse L cells or frog oocytes (Pellicer et al., 1978; McKnight and Gavis, 1980); this expression is mediated by four cis-acting elements that correspond to the binding sites of cellular transcription factors, including a proximal TATA box and three upstream regions, where two Sp1 binding sites are separated by a CAAT box (McKnight, 1982; McKnight and Kingsbury, 1982; McKnight et al., 1984; Jones et al., 1985). However, in viral infection, efficient expression of the functional TK gene must be regulated by the IE protein and depends on the transcriptional mechanism of cellular RNA polymerase II (Pol II) throughout the transcription process (Wagner and DeLuca, 2013).

ICP0, ICP4, and ICP27 are believed to be the major regulatory proteins of HSV-TK gene transcription, especially ICP4, which is required for transcriptional activation of the TK gene (Mackem and Roizman, 1982; Yu et al., 2010). The HSV-TK gene can be induced by ICP4 in the absence of upstream Sp1 and CAAT binding sites, even if the TATA box is disrupted. In fact, these four cis-acting elements are mainly related to the efficiency of TK gene expression and appear to be time independent of transcription initiation. Transcriptional activation of the HSV-TK gene by ICP4 is accomplished by the transactivation mode of transcription, which does not require these elements (Imbalzano et al., 1991; Imbalzano and DeLuca, 1992; Cook et al., 1995). ICP27, in addition to its established role as a posttranscriptional regulator of TK gene expression, may also modulate the DNA binding activity of ICP4 by affecting its posttranslational modification status; co-immunoprecipitation experiments show the inability of ICP4 from the extracts of infected cells to bind a TK probe in the absence of ICP27 (Panagiotidis et al., 1997). ICP0 has a great influence on the transcription rate of the whole virus E genome, and the inactivation of ICP0 can significantly downregulate the mRNA levels of the early TK enzyme and ICP6 in the absence of ICP4 (Samaniego et al., 1997).

## Structure and function of α-Herpesviruses Thymidine Kinases

HSV-1 TK, encoded by the UL23 gene, is composed of 376 amino acids (aa) (McKnight, 1980). The amino acid sequence of the viral TKs of different herpesviruses is not conserved as a whole, but there are six active or highly conserved gene regions (in HSV-1): aa 51–63 (I), aa 83–88 (II), aa 162–164 (III), aa 168–176 (IV), aa 216–222 (V), and aa 284–289 (VI) (Sauerbrei et al., 2016). Regions I and III, the most conserved core regions associated with TK activity, are the ATP-binding site and the substrate-binding (thymidine-recognition) site, respectively. Region I involves a segment (-GXXGXGK-) co-existing in most herpesvirus TKs analogous to the “glycine loop”; the flexible structure folded from the corresponding peptide can accommodate the β-phosphate of ATP in many ATP-binding proteins, such as the sequence of aa 15–21 (-GGPGSGK-) in viral adenylate kinase (AK) (Tsai and Yan, 1991; Schulz, 1992). In addition, the hydrophobic pocket conformation in this region is extremely advantageous for the binding of the ATP adenine ring; when any of the three glycine (Gly) residues in the motif are mutated, the enzyme activity is completely lost (Liu and Summers, 1988). The ATP-binding site has high specificity, but it also has a high affinity for GTP, CTP, TTP, and their corresponding deoxygenated forms when the ATP content is extremely low (Gentry, 1992; Wild et al., 1997). The conserved aspartate (Asp) residue in position III (-DRH-) is believed to participate in nucleotide binding because the position of this residue is very similar to the position of Asp in the binding site of various kinases to Mg^2+^-ATP (the front has a hydrophobic β-sheet) (Robertson and Whalley, 1988). In fact, this Asp residue is a ligand that binds both Mg^2+^ and NTP; the Asp residue depends on two water molecules to coordinate Mg^2+^ between the β- and γ-phosphates of ATP, and the complex of the ATP molecule and Mg^2+^ binds to the nucleotide binding site (Abele and Schulz, 1995; Wild et al., 1997). Region V (-RXXXRXR-) is a lid domain rich in arginine (Arg) residues; positive charge clusters and Mg^2+^ ions from this domain make phosphorus atoms susceptible to nucleophilic attacks by deoxyribose 5’-OH polarized by Glu_225_ and Glu_83_ (in HSV-1), which in turn activates the γ-phosphate and deoxyribose 5’-OH of ATP to participate in catalytic reactions (Wild et al., 1997). Region VI is considered to be involved in the deoxyadenosine kinase (dTMPK) activity of HSV-TK, and combining deoxyadenosine (dTMP) with the substrate recognition site containing the -DRH- motif and the terminal residue before the glycine ring involves deoxyadenosine (Gentry, 1992).

HSV-1 TK exists in the form of a homodimer; it is very similar to human TK1 and can be classified as a homologue of TK1, but HSV-1 TK has a broader substrate specificity than human TK1 and is essentially a polynucleotide kinase (Johansson et al., 2001; El Omari et al., 2006). The kinase activity of TKs is determined by their structure; for example, HSV-1 TK is an α/β structure composed of 15 α-helices and 7 β-sheets whose core folding structure has a parallel five-strand β-sheet similar to that of deoxyribonucleoside kinase (dNK) and deoxyguanosine kinase (dGK) but with an antiparallel β-chain and an α-helix in the C-terminal and an additional domain comprised of approximately 65 amino acid residues (between aa 197–198 in dNK and aa 236–237 in dGK) located opposite to the active site on the subunit, which extends the subunit interaction region in HSV-1 TK (Brown et al., 1995; Johansson et al., 2001). Based on the mimesis of natural nucleoside molecules, nucleoside analogues such as the thymidine analogue BrdU and purine nucleoside analogues such as ACV and ganciclovir (GCV) are recognized by HSV-1 TK, since the triphosphate form of these compounds is a substrate for viral and/or cellular DNA polymerases; thus, these compounds are widely used in antiviral therapy (Deville-Bonne et al., 2010). However, the activity and substrate specificity of γ-herpesvirus TKs appear different from those of α-herpesvirus, especially HSV-1, because neither EBV-TK nor HHV-8 TK can phosphorylate GCV, and the substrate specificity of HSV-1 TK is broader than that of other viral TKs (Gentry, 1992; Gustafson et al., 1998, 2000; Lock et al., 2002). In addition to its important activity as an HSV-1 TK, the protein encoded by the UL23 gene seems to be a component of the mature virion tegument. Loret et al. found *via* biochemical assays that UL23 proteins are indeed present in mature extracellular virions; moreover, this protein shares properties with the VP16 tegument, suggesting strongly that UL23 proteins are true components of the HSV-1 viral tegument (Loret and Lippé, 2012).

## Effect of Thymidine Kinase Genes on α-Herpesvirus Virulence and Latent Infection

### Effect of Thymidine Kinase Genes on α-Herpesvirus Virulence

Initially, the biological functions of HSV-TK were not very clear, but early studies noted that HSV-TK expression appeared to be dispensable for HSV infection in cell culture (Dubbs and Kit, 1964). Jamieson et al. first suggested a possible biological role for HSV-TK by noting that although TK expression does not affect HSV infection in dividing cells *in vitro*, it is necessary for viral replication in nondividing cells or serum-starved cells in culture (Jamieson et al., 1974). In fact, many gene functions that appear to be nonsignificant in cultured cells or tissues are extremely important for viral infection with complex cell-virus interactions *in vivo*.

In 1978, Field and Wildy noticed that the TK gene plays a vital role in HSV virulence, as the severity of neurological symptoms and the rates of death decreased dramatically following either intracerebral or peripheral inoculation of mice with TK-mutants of HSV-1 and HSV-2, which fail to induce TK, compared with the effects of their respective parent strains (Field and Wildy, 1978). Several continuous studies confirmed decreased virulence, i.e., decreased mortality, after inoculation of animals with many different HSV-TK mutants (Stroop et al., 1994; Huang et al., 2017; Omura et al., 2017). Additionally, other TK-mutants of α-herpesviruses in different subfamilies, even some TK-defective DNA viruses, have very similar virulence features (Mittal and Field, 1989; Sanford et al., 2016; Dong et al., 2017). Recently, two assessment reports about the virulence of PRV-TK-mutants in susceptible animals that note that triple gene-deletion PRV gE/gI/TK-mutants are less pathogenic to pigs and avirulent to mice and sheep than double gene-deletion PRV gE/gI-mutants and the highly virulent parent strains (Cong et al., 2016; Dong et al., 2017). As may be inferred from those findings, the TK gene is the critical virulence gene of α-herpesvirus. In principle, virus virulence depends on various host and viral factors; as predicted by Jamieson et al. (Jamieson et al., 1974), due to nondividing neurons with very low thymidylic acid metabolism levels that may be comparable to those of serum-starved cells or resting cells, the ability for viral TK expression is quite important for viral replication *in vivo* infections. This reason may acceptably explain the reduction in the neurovirulence of these TK-mutants to some extent. Some research supports the idea that cellular TK has a positive effect on virus growth and the maximum expression of virus virulence (Suzutani et al., 1995), and most cellular TK can functionally replace the specific viral TK to promote HSV replication in mouse sensory nerves (Chen et al., 1998). In addition, providing viral TK by mixed infection with wild-type virus can also increase the infection of the mouse trigeminal ganglion (Tenser and Edris, 1987; Coen et al., 1989).

### Effect of Thymidine Kinase Genes on Latent α-Herpesvirus Infection

During primary infection in the host, the replication of HSV-1 generally starts in peripheral tissues such as eye, and then the virus spreads to peripheral sensory ganglia, such as trigeminal ganglia (TG), and the central nervous system (CNS) before establishing lifelong latency maintained by the presence of viral genomes in neurons (Mingo et al., 2012). By using various TK-mutants, *in vitro* studies showed that the TK gene is nonessential for α-herpesvirus replication in cells, including sensory neurons cultured from dorsal root ganglia of rat embryos (Kennedy et al., 1983; Wilcox et al., 1992; Wu et al., 2017). However, in vivo work indicated that HSV-TK expression is indispensable for neuronal infection and latency (Field and Wildy, 1978; Tenser et al., 1979; Tenser and Dunstan, 1979; Chen et al., 2004; Huang et al., 2017). So far, the mechanism of α-herpesvirus latency influenced by the TK gene is still incompletely clear. Specifically, what role does the TK gene plays in the stage of latency: establishment or reactivation?

A few researchers believed that the viral TK gene is required for the efficient establishment of latency, because the replication of the HSV TK-mutant is severely restricted in ganglia, and cell-to-cell spread of virus is not detected within ganglia (Thompson and Sawtell, 2000). In fact, this opinion is contrary to most previous studies. In early molecular biology research, Ho and Mocarski inserted a modified *Escherichia coli* lacZ gene under the control of HSV α4 or β8 regulatory signals into the HSV-1 genome, disrupting the viral TK gene; by using β-galactosidase as an *in situ* indicator of viral gene expression, they found evidence that the virus could be detected in ganglion neurons, as β-galactosidase expression was detected in ganglia after the peripheral inoculation of mice with TK-deficient viruses (Ho and Mocarski, 1988). The most direct and hard evidence comes from the discovery of HSV latency-associated transcript (LAT). The noncoding LAT, transcribed from within the long repeats of the viral genome, is the most abundant viral transcript during latency of both HSV-1 and HSV-2 (Stevens et al., 1987). Compared with other viral promoters, the LAT promoter is highly active during latency, and LAT is the only viral gene product that is readily detectable during latency (Kawamura et al., 2018). Tenser et al. showed that virus is isolated from 0% of ganglia after inoculation with HSV TK-mutants, but LAT is detected in 95 to 100% of ganglia (Tenser et al., 1989). Leist et al. suggested that HSV TK-mutants could establish latent infections with near wild-type efficiency in mouse dorsal root ganglion (DRG) because the number of neurons expressing LAT RNAs was only slightly reduced following footpad inoculation (Leist et al., 1989). Work by Coen et al. confirmed that LAT-positive neurons can also detected following corneal inoculation with TK-mutants (Coen et al., 1989). Based on these observations, HSV TK-mutants do establish latent infections, and the ability of these viruses to establish latency seems to be uncorrelated to the efficiency of expression of the TK gene (Sears et al., 1985).

Actually, the TK gene is important for the reactivation of virus latency. Coen et al. showed that although viral DNA cannot proliferate in neurons, it remains bioavailable within the nucleus, as HSV-TK-mutants can be activated from neurons in secondary overlapping infections with wild-type virus, but reactivation of the HSV-TK-mutant alone is defective (Coen et al., 1989). Efstathiou et al. *via* Southern blot hybridization confirmed *in vivo* complementation of the TK-mutant by wild-type virus in both peripheral and central nervous system tissues of mice during acute infection and showed that such complementation can result in the reactivation of latent TK-infection (Efstathiou et al., 1989). Not only complementation but also competition is between the HSV TK-mutant and wild-type HSV during co-infection of mouse trigeminal ganglia. Chen et al. found that wild-type virus do not always complement the acute replication or increase the number of latent viral genomes of the TK-mutant in mouse ganglia; even so, wild-type virus can still confer the pathogenic phenotype to TK-mutants, somehow providing sufficient TK activity in trans to permit a TK-mutant to reactivate from latently infected ganglia (Chen et al., 2006).

## Progress in the Functional Application of α-Herpesvirus Thymidine Kinase Genes and Thymidine Kinases

### Antiviral Treatments

**Figure 2:**
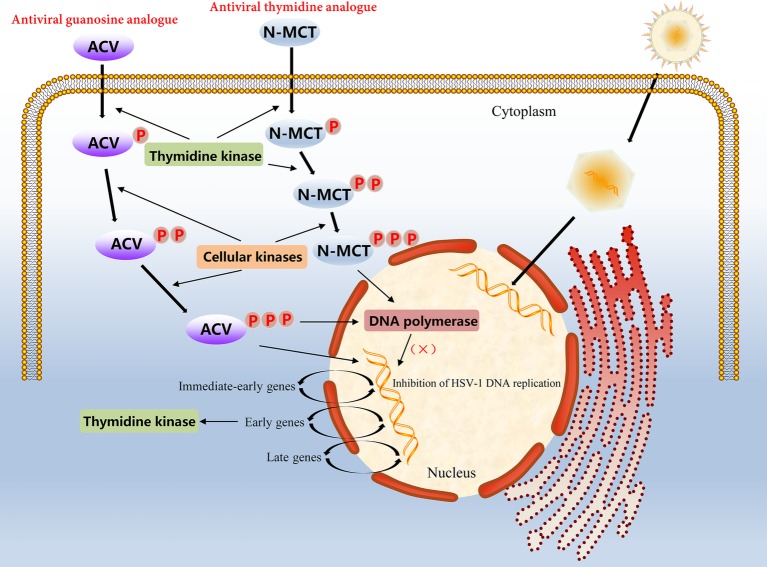
Molecular mechanism of action of the antiviral guanosine analogue ACV and the thymidine analogue N-MCT in HSV-1 infected cells.

In α-herpesvirus-infected cells, the level of viral TK is very high because of the rapid proliferation of the virus; therefore, certain nucleoside analogues, such as ACV, can be taken up into cells to achieve antiviral effects. However, ACV is not a direct antiviral agent; it has to undergo three phosphorylation stages to play an antiviral role. ACV must first be phosphorylated by the viral TK into an intermediate product, ACV-monophosphate (ACV-MP). Next, ACV-MP is subjected to the two remaining phosphorylation steps by endogenous kinases to generate the final product, ACV-triphosphate (ACV-TP), which is a substrate for DNA polymerase that can be incorporated into the 3′ end of the DNA synthesis strand and competitively hinder the binding of deoxynucleoside triphosphates to DNA extensions during replication (Miller and Miller, 1980, 1982; Elion, 1993). Because ACV-TP at the 3′ end does not have a hydroxyl group, it cannot be cleaved by 3′ → 5′ exonucleases, which makes the blocking effects of ACV-TP on DNA synthesis quite noticeable (Derse et al., 1981). In addition, ACV-TP is an inactivator of the herpesvirus DNA polymerase and has a weak reversible inhibitory effect on host DNA polymerase α, but the minimum inhibitory concentration against the latter is approximately 3,000 times higher than that against the former (Figure 2; Miranda et al., 1979; Furman et al., 1984). Based on the broad substrate specificity of HSV-TK, other nucleotide analogues such as valacyclovir (VCV), penciclovir (PCV), and famciclovir (Famvir) can also be used as antiviral drugs, but these nucleoside analogues perform differently in terms of bioavailability, potency, tolerance, and drug toxicity (James and Prichard, 2014). For example, PCV is not an obligate DNA synthesis terminator, because its side chain has a 3′ hydroxyl group, which allows DNA strands to continue to extend during replication (Piret and Boivin, 2011). Furthermore, the oral availability of PCV is extremely poor, and Famvir is often used as a diethyl ester prodrug to improve bioavailability. Most important of all, these antiviral drugs have no significant inhibitory effect on viral replication in latency and mainly target the synthesis of viral DNA during the lytic replication period (Skoreński and Sieńczyk, 2014).

Antiherpesvirus drugs have been developed for more than 60 years, and ACV and its derivatives constitute the first-line therapy for the management of HSV infections. However, long-term prophylactic and curative ACV treatments may cause the emergence of HSV resistance, and the prevalence of HSV resistance to ACV has been reported to vary from 3.5 to 10%, with higher rates in immunocompromised individuals and hematopoietic stem cell transplant recipients (James and Prichard, 2014; Skoreński and Sieńczyk, 2014). In up to 95% of cases, ACV-resistant HSV isolates are clinically associated with mutations in the viral TK gene, and overall, the nucleotide mutation rate of the HSV-1 TK gene appears to be higher than that of the HSV-2 TK gene (Burrel et al., 2010). Typically, those selective mutations in the TK gene result in three manifestations: low expression of viral TK, no expression of active TK, and altered substrate catalytic properties of TK so that ACV is incapable or only partially capable of phosphorylation (Piret and Boivin, 2011; Schubert et al., 2014). According to the statistics in a sample studied by Sauerbrei et al., among the 134 different mutations linked to drug resistance detected on the HSV-1 TK gene, 63.0% were identified as nucleotide substitutions causing amino acid changes, 35.6% were attributed to frameshift mutations, and 1.4% were due to base insertions or deletions; the proportions of these mutation types were 40.3, 52.8 and 6.9% in 72 HSV-2 TK gene mutation samples, respectively (Sauerbrei et al., 2016). Although these mutations were mainly located outside the conserved region of the genome, all the corresponding HSV-TK mutants were found to be resistant to ACV.

Growing concerns relative to drug-resistant mutations in α-herpesvirus strains make evident the require of other compounds with significant activity of virus resistance. For example, another thymidine analogue N-methanocarbathymidine (N-MCT) has been used in a variety of live animal experiments of HSV-2 infection and has shown better antiviral effects than high-dose ACV therapy in preclinical evaluation (Bernstein et al., 2011, 2015; Quenelle et al., 2011). Although the accumulation rate of N-MCT and GCV phosphate in HSV-1 infected cells was similar, the decay rate of N-MCT-TP was slower than that of GCV-TP. The metabolic pathway of N-MCT is slightly different from that of ACV; N-MCT has to be phosphorylated twice by HSV-TK, and then the intermediate needs to be additionally phosphorylated by the cellular kinase to be phosphorylated into the triphosphate form product, which finally acts on the target virus DNA polymerase (Zalah et al., 2002; Figure 2). Additionally, several helicase-primase inhibitors (HPIs) unrelated to the nucleoside analogues has also drawn attention, including pritelivir and amenamevir. Pritelivir was observed no drug-resistance after 4 weeks daily therapy of 155 patients with HSV-2 associated genital lesions (Edlefsen et al., 2016). Recently, a phase II clinical trial on the safety and efficacy of pritelivir for ACV-resistant HSV infection in immunocompromised patients (PRIOH-1) is also initiated by AiCuris Anti-Infective Cures Gmbh (NCT03073967). For amenamevir, the investigation has completed a clinical study on herpes zoster, and the drug has been used for the treatment of herpes zoster in Japan (Shiraki, 2018). Due to the use of antiviral drugs usually leads to emergence of drug-resistance associated mutations, more efforts need to be putted in the discovery of new viral targets.

### Antitumor Therapy

**Figure 3:**
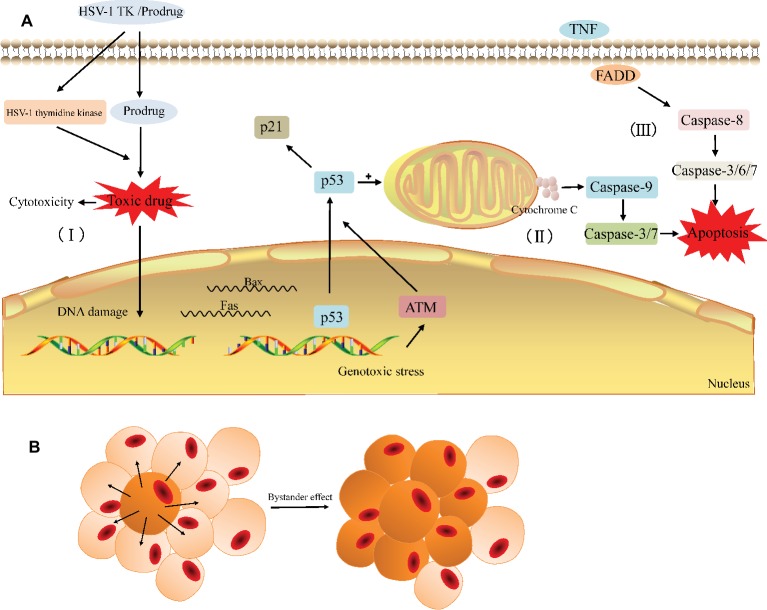
General principle of the HSV-TK suicide gene system. **(A)** The HSV-TK/prodrug system induced cytotoxicity and apoptosis in tumor cell. (I) The HSV-1 TK gene coding for thymidine kinase is delivered to the target cell. The expression of HSV-1 thymidine kinase and cellular kinases allows a prodrug (GCV) to be activated as the toxic drug (GCV-TP) in the cell and damage the DNA of the target cell. (II) Gene therapy induces endogenous apoptosis by activating p53 signaling pathway in the target cell. (III) Gene therapy induces exogenous apoptosis. **(B)** The bystander effect induces the death of adjacent cells.

The HSV-TK/GCV system is one of the most promising gene therapies for the tumor. After HSV-TK gene was transfected into tumor cells, its encoding product (thymidine kinase) can phosphorylate non-toxic GCV to cytotoxic GCV triphosphate (GCV-TP), which is involved in the DNA replication and leads to an advanced replicate termination with the following apoptosis of cancer cells (Figure 3; Chen et al., 2014; Zeng et al., 2014). As the affinity of GCV to HSV-TK is about 1,000 times higher than that of cell TK, GCV is preferentially phosphorylated by exogenous HSV-TK in TK-positive tumor cells (Osaki et al., 1994); in other words, the HSV-TK/GCV system has little effect on normal tissues while performing its antitumor role.

In general, the possible pathways *via* which the HSV-TK/GCV system induces tumor cell death include apoptotic and nonapoptotic pathways, but the specific mechanism has not yet been fully elucidated (Wei et al., 1998; Tomicic et al., 2002b; Yin et al., 2012; Zeng et al., 2014). The non-apoptotic pathway is primarily involved in the direct cytotoxicity. Cytotoxic nucleoside analogues can result in cytoskeletal reorganization and various types of cellular DNA damage, such as base pairing errors, irreparable DNA double-strand breaks (DSBs), sister chromatid exchanges (SCEs), and lethal gene destabilization (Halloran and Fenton, 1998; Tomicic et al., 2002a; Ladd et al., 2011; Chen et al., 2014; Zeng et al., 2014). Ladd et al. found that cytotoxicity depends on the degree of GCV phosphorylation and the cytotoxic effect increased significantly when the homologous recombination repair (HRR) of the damaged gene is blocked (Ladd et al., 2011, 2013). In the apoptotic pathway, the activation of the p53 gene and p53 signaling pathway in tumor cells appears to be critical. Beltinger et al. demonstrated that HSV-TK/GCV treatment induces p53 accumulation and the cell surface expression of CD95 and the TNF receptor in human neuroblastoma cells. p53 can mediate the translocation of CD95 to the cell surface and CD95 aggregation, leading to the formation of a death-inducing signaling complex containing the FADD and caspase-8 to induce apoptosis (Beltinger et al., 1999). Next, Beltinger and colleagues also noted that treatment with HSV-TK/GCV leads to mitochondrial perturbations, including the loss of the mitochondrial membrane potential and the release of cytochrome c from mitochondria into the cytosol, thus inducing nuclear fragmentation and caspase activation (Beltinger et al., 2000). The perturbation of mitochondrial function can mediate p53 protein accumulation and regulate the effector phase of apoptosis to amplify TK/GCV-induced apoptosis. Zeng et al. showed that the protein expression of p53 increases with HSV-TK/GCV treatment and that the expression of p21 is upregulated through a p53-dependent DNA damage signaling pathway, leading to a decrease in the protein expression of PCNA, cyclin B, and CDK1 and promoting cell cycle arrest in human breast cancer cells (Zeng et al., 2014). Additionally, Liu et al. also confirmed showed that HSV-TK/GCV therapy is at least partially dependent on p53 status in tumor cells evident as the expression of exogenous p53 and the overexpression of ASPP2 that interacts with endogenous p53 can enhance HSV-TK/GCV-induced cell death in HCC cells lacking functional p53 (Liu et al., 2016). In summary, HSV-TK/GCV-induced tumor cell apoptosis involves both endogenous and exogenous apoptotic pathways (Figure 3A).

Similar to other suicide gene systems, the injurious effect of the HSV-TK/GCV system on tumors largely depends on the unique “bystander effect” (Figure 3B; Moolten, 1986). Among the mechanisms involved in bystander effect, the simple diffusion of toxic products (Hirschowitz et al., 1995), local anemic necrosis of tumors (Ram et al., 1994), secondary apoptosis of bystander cells (Freeman et al., 1993; Hamel et al., 1996), and local specific active immunity (Caruso et al., 1993; Vile et al., 1994) are possibly related to this effect. However, more evidence suggested that the bystander effect has the close correlation with the gap junctional intercellular communication (GJIC). Gap junction (GJ) channels directly facilitate the intercellular exchange of ions and small molecules including the toxic metabolites of GCV, which pass directly from HSV-TK expressing cells to surrounding cells that do not express it, so the bystander effect in suicide gene therapy is usually proportional to the degree of GJIC in cancer (Mesnil and Yamasaki, 2000; Matono et al., 2003; Xiao et al., 2017). Gap junctions are essentially clusters of transmembranous channels composed of hexamers of connexin (Cx), which provide a pathway for the diffusion of ions and small molecules between adjacent cells (Beyer and Berthoud, 2018). Manipulating the expression of Cx can greatly affect GJIC and the bystander effect and then affect the efficacy of HSV-TK/GCV therapy. For example, the generation of increasing free Cx43 levels is essential for reconstructing GJIC to increase GCV permeability and the bystander effect of HSV-TK/GCV gene therapy (Xiao et al., 2017; Zhang et al., 2018). Nicholas et al. showed that enhancing Cx43 expression in low GJIC HT-29 colorectal tumor cells increases bystander cell death and reduces tumor burden beyond what is expected from HSV-TK/GCV treatment alone (Nicholas et al., 2003). Paíno et al. found that the up-regulation of Cx43 increased the bystander effect in HSV-TK/GCV therapy against human glioma cells and this effect was impaired in the presence of inhibitors of GJIC (Paíno et al., 2010). Furthermore, a number of classes of chemicals, including gemcitabine (Garcia-Rodriguez et al., 2011) and all-trans-retinoic acid (ATRA) (Wu et al., 2016), have been reported to increase Cx26 and Cx32, respectively, and subsequently enhance GJIC and the bystander effect. From these results, Cx and Cx-mediated GJ can be therapeutic targets in cancer to enhance the efficacy of suicide gene therapy for cancer.

In the past decades, the effectiveness of the HSV-TK/GCV suicide gene system in antitumor therapy has been acknowledged through a variety of tumor model mice (Moolten and Wells, 1990; Ram et al., 1993; O’Malley et al., 1995; Hall et al., 1998). However, this approach is limited in part by some problems associated with high ganciclovir (GCV) doses used to achieve tumor cell killing. For example, high GCV dose could result in suppression of immune system and damage to bone marrow cells (Kokoris and Black, 2002; Malekshah et al., 2016). On the other hand, the poor cellular uptake of GCV into the tumor cells may be an important reason for the limited efficacy of HSV-TK/GCV gene therapy. *In vitro*, Rubsam et al. found that the accumulation of phosphorylated GCV-TP in 20–80% bystander cells of human glioblastoma cells expressing HSV-TK occurred readily, but this accumulation was dependent upon the percentage of HSV-TK-expressing cells, as well as the concentration of GCV and the length of incubation (Rubsam et al., 1999). According the result of Sandmair et al., ≥ 10% transfection efficiency is required for a successful reduction in BT4C glioma tumor size with HSV-TK/GCV treatment *in vivo* (Sandmair et al., 2000). Additionally, Haberkorn et al. showed that the uptake of GCV is correlated closely with the growth inhibition (Haberkorn et al., 1998). The sensitivity toward GCV varied in different tumor cell lines, and the cellular uptake of GCV in some tumor cells is poor (Beck et al., 1995). Gynther et al. showed that GCV uptake into BT4C glioma cells *in vitro* at 100 μM was only 2.1 pmol/mg of protein, corresponding to only 0.12% of the extracellular concentration could be get into BT4C cells. In their BT4C rat glioma model, the areas under the concentration curve of unbound GCV in blood, brain extracellular fluid (ECF), and tumor ECF were 6,157, 1,658, and 4,834 μM·min, respectively. The apparent maximum unbound concentrations achieved within 60 minutes were 46.9, 11.8, and 25.8 μM in blood, brain, and tumor, respectively. The unbound GCV concentrations in brain and tumor after *in situ* rat brain perfusion were 0.41 and 1.39 nmol/g, respectively (Gynther et al., 2015). In human, GCV is also poorly absorbed after oral administration, with a bioavailability of approximately 5% (Mareri et al., 2016). The poor uptake into the tumor cells is probably due to the high polarity of GCV, which hinders its permeability across cell membranes. But despite these limitations, HSV-TK/GCV system was successfully used in few clinical trials (Voges et al., 2003; Li et al., 2007; Nasu et al., 2007; Putten et al., 2010; Sangro et al., 2010; Chiocca et al., 2011). Most of these clinical trials are in phase I/II, and adenoviral vectors are the preferred vector for the HSV-TK system, especially for liver cancer treatment, possibly because of their hemorrhagic properties, high efficiency, and ability to grow to high titer. The results showed that Ad-TK/GCV therapy, as an adjunct to conventional chemotherapy, could significantly improve the clinical treatment effect and prognosis and reduce the recurrence rate in patients with liver cancer (Li et al., 2007). The Ad-TK/GCV system is well tolerated for the treatment of liver cancer because no serious side effects were observed in patients receiving up to 2 × 10^12^ virions, and the tumors remained stable and even developed necrotizing signs in some patients (Sangro et al., 2010). A phase III clinical study demonstrated a significant positive effect of an adenoviral vector encoding the HSV-TK treatment on time to reintervention or death when compared with standard care treatment (Putten et al., 2010). At present, high-efficiency and less-immunogenic third-generation adenoviral vectors have been introduced, and the successful development of new recombinant viral and non-viral vectors, such as mesenchymal stem cells and nanoparticle carriers, will strongly promote the progress of suicide gene systems in clinical trials (Nouri et al., 2015; Gao et al., 2016).

To promote the therapeutic efficiency and safety, as well as to minimize the side effects of different treatments, combining suicide genes with various traditional and classic therapeutic modalities is being very attractive. Luo et al. showed that combination treatment with PHSP-TK plus radiofrequency hyperthermia (RFH) results in higher TK gene transfection/expression in vitro and *in vivo* (Luo et al., 2016). In cholangiocarcinoma bearing mice, interventional RFH treatment significantly promotes the inhibitory effects of PHSP-TK/GCV therapy evident as in vivo significant reduction in tumor size and higher apoptosis index (Luo et al., 2018). Another experiment in tumor-bearing mice or rats also showed that RFH-enhanced HSV-TK/GCV gene therapy for non-small-cell lung cancer is feasible (Ji et al., 2018). Chen and Tang found that the HSV-TK/GCV system in combination with radiotherapy also has stronger therapeutic effects on the tumor. In their nude mice models, the inhibition rate of transplanted human cervical cancer cell by HSV-TK/GCV suicide gene therapy system alone and radiotherapy alone was 39.5 and 35.8% in vivo, respectively, but the inhibition rate of combination therapy was 87.9% (Chen and Tang, 2010). In clinical trials, the HSV-TK suicide gene therapy in combination with surgery and accelerated radiation showed an increase in patient survival rates in malignant glioma patients (Chiocca et al., 2011). There were also reports that HSV-TK gene-mediated cytotoxic immunotherapy as adjuvant to surgery or chemoradiation for pancreatic adenocarcinoma in pancreatic, combination therapy increased the modern chemoradiation efficacy adenocarcinoma without added toxicity (Aguilar et al., 2015). Currently, a few clinical trials are under way to assess the efficacy and toxicity of HSV-TK/VCV gene therapy in combination with radiotherapy in recurrent glioblastoma multiforme or anaplastic astrocytoma (NCT03596086) and the efficacy and toxicity of HSV-TK/VCV gene therapy in combination with androgen deprivation therapy, brachytherapy, external beam radiotherapy, and prostatectomy in previously untreated high-risk prostate cancer (NCT03541928).

The HSV-TK/GCV system also can be used in combination with some potential therapeutic targets in order to enhance its anticancer efficacy. Gong et al. found that numerous genes encoding immune-inflammatory response and genes encoding inflammatory response related proteins are upregulated in spontaneous HCC of the HSV-TK transgenic mice, and some genes involved in DNA replication and cell cycle-related genes and master regulators of cell cycle checkpoint signaling pathways are also induced in tumor (Gong et al., 2018). Indeed, researchers generally consider that chronic inflammation is an essential underlying condition in the occurrence and development of tumors. Specifically, inflammation in the tumor microenvironment has the following positive effects on tumors: promoting the proliferation and development of tumor cells, promoting new angiogenesis and metabolism and increasing vascular permeability, destroying adaptive immune responses, changing the response of tumors to hormones and chemotherapeutic drugs, and promoting the infiltration and metastasis of tumor cells (Allavena et al., 2008; Sappayatosok et al., 2009; Jesmin et al., 2012; Kanda et al., 2017). Activation of some transcription factors such as NF-κB and STAT3 is frequently detected in tumors. NF-κB is necessary to promote the survival and proliferation of cancer cells by inducing the expression of anti-apoptotic genes (BCL-2) and activating cyclin, and activation of STAT3 in tumor cells enhances the ability of tumors to escape the immune system (Coussens and Werb, 2002; Jeyasuria et al., 2011). Rather, several studies showed that inflammation is also associated with the inhibition and remission of tumors. For example, in a skin tumor model, the overexpression of NF-κB inhibits invasive epidermal tumors (Dajee et al., 2003). Innate immunity is very important to the initiation of adaptive immunity that has the effect of immune surveillance and elimination of new growing tumor (Li et al., 2019). Kuriyama et al. found that cancer gene therapy with HSV-TK/GCV system depends on T-cell-mediated immune responses and causes apoptotic death of tumor cells in vivo (Kuriyama et al., 1999). Based on this close and complex relationship between immune inflammation and inflammation-induced cell cycle activation and cancer, inflammatory factors and pathways can be as potential targets in combination with HSV-TK/GCV suicide gene therapy in antitumor therapy. Several cell cycle–related genes (CCNE1 and GADD45) (Abate-Daga et al., 2010) and immune genes (IL-18) (Higashi et al., 2014) are functionally validated as conditioners of cellular sensitivity to HSV-TK/GCV. Ahn et al. showed that silencing of STAT3 can enhance the antitumor activity of HSV-TK delivered by an adenoviral vehicle by inhibiting cell proliferation and eliciting an anticancer immune response in tumor-bearing mice (Ahn et al., 2012). Similarly, STAT3/NF-κB-regulated HSV-TK/GCV suicide gene therapy has a positive effect on cisplatin-resistant triple-negative breast cancer in mouse model (Kuo et al., 2017). With the deepening of the research on the relationship between inflammation and cancer, some new therapeutic methods and new therapeutic targets will be applied to cancer prevention and treatment.

### Regulation of Stem Cell Transplantation

The HSV-TK/GCV system is a novel therapeutic strategy for the modulation of graft-versus-host disease (GVHD), a major complication of allogeneic stem cell transplantation (allo-SCT). In clinical trials, donor T-lymphocytes expressing HSV-TK has been used in a phase I/II study of allogeneic bone marrow transplantation to address rejection caused by T lymphocytes (Tiberghien et al., 1997). Efficacy study on the strategy of HSV-TK engineering donor lymphocytes to treat patients with high risk acute leukemia is underway (NCT00914628). Equally important, this system is required to provide an “emergency exit” switch to regulate the differentiation of transplanted cells and the removal of residual undifferentiated cells to avoid the abnormal localization, non-directional differentiation or hypertrophy of transplanted cells, or the formation of teratoma during stem cell or cell derivative transplantation (Sułkowski et al., 2018).

Early research has shown that undifferentiated human embryonic stem (ES) cells transfected with the HSV-TK gene can maintain a normal karyotype and multidirectional differentiation ability, but the resulting sensitivity to GCV limits the proliferation and differentiation activities of these cells, and this effect is more obvious for early- or high-dosage regimens (Schuldiner et al., 2003; Naujok et al., 2010). In all related studies, GCV only targets the selective destruction of undifferentiated cell populations, and differentiated cells inactivated by the promoter are not affected; this selectivity is usually ascribed to the transcription of the HSV-TK gene only in undifferentiated cells (Ou et al., 2013). The regulation strategy of the HSV-TK/GCV system is applicable to the transplantation process of not only ES cells but also other transplantable cells, such as neural stem cells (NSCs) and induced pluripotent stem cells (iPSCs) (Tieng et al., 2016; Sułkowski et al., 2018). However, some researchers observed that the characteristics of HSV-1 TK+ ES cells surviving after GCV treatment may be abnormal; for example, the transcription of intracellular growth-regulating factors such as SRY-like HMG box 2 (Sox2) was susceptible in cells, and those cells even exhibited neuron-like differentiation (Hara et al., 2008; Naujok et al., 2010). A recent study provided a warning against using the HSV-TK gene in human iPSCs, particularly in clinical applications, because high-level and/or constitutive expression of HSV-TK resulted in the induction of cell death or silencing of HSV-TK expression. Furthermore, excessive accumulation of thymidine triphosphate, caused by HSV-TK expression, resulted in an imbalance in the dNTP pools, and this unbalanced state led to DNA synthesis inhibition and cell death in a process similar to a “thymidine block” (Iwasawa et al., 2019). For these reasons, its reliability and the security are waiting for further solving.

### Reporter Genes

As discussed above, the HSV-TK gene plays important roles in antiviral treatments and in antitumor therapy because of the broad substrate specificity. However, the structural basis for this broad substrate recognition is not completely clear. Due to clinical isolates of α-herpesviruses resistant to ACV are increasing in frequency (Burrel et al., 2010; Sauerbrei et al., 2016), this structural information is critical for designing TK molecular models and improving nucleoside drugs. Additionally, HSV-1 TK is as the suicide gene in antitumor therapy, a better understanding of the molecular mechanism of HSV-TK could lead to future customized, drug-specific TKs for gene therapy. Photo-affinity labeling (PAL), or photocrosslinking, is an efficient and reliable tool to identify, isolate, and characterize novel biological molecules and potential drug targets and study protein–protein interactions in the complex proteome (Murale et al., 2016). For example, photoaffinity labeling with two photo-affinity analogs [α-^32^P]5-azido-2′-deoxyuridine-5′-monophosphate ([α-^32^P]5-N_3_dUMP) and [γ-^32^P]8-azidoadenosine-5′-triphosphate ([γ-^32^P]8-N_3_ATP) was used by Rechtin et al. to characterize the thymidine, thymidylate, and ATP active sites of the HSV-1TK (Rechtin et al., 1995). In 1996, they *via* these photo-affinity analogs confirmed that the thymine base of thymidine and TMP bind at one shared site in HSV-1 TK (Rechtin et al., 1996). Despite the significance, few photocrosslinkers are currently available the HSV-TK.

Not only that but also exploring intracellular behavior of the HSV-TK suicide gene is necessary for improving the efficacy and safety in cancer therapy. Various studies have indicated that the utilization of molecular imaging is an essential tool for monitoring tumor response to various therapies/drugs, gene expression, and tracking therapeutic cells (Raty et al., 2007; Bensch et al., 2013). In general, imaging modalities mainly includes positron emission tomography (PET), single photon-emission computed tomography (SPECT), X-ray computed tomography, bioluminescence, ultrasound, and magnetic resonance imaging (MRI). In indirect tracking, nucleoside analogues labeled with radionuclides (^18^F, ^124^I, ^131^I, ^14^C, etc.) is frequently used as specific probes for the HSV-TK gene in SPECT or PET imaging (McCracken, 2018). These labeled molecules can be phosphorylated to polar 5′-phosphate nucleosides by HSV-TK after they are actively transported into HSV-TK transfected cells, and according to the degree of intracellular radioisotope labeling, probe enrichment can directly reflect the expression of the HSV-TK gene and TK enzyme activity (Tian et al., 2012). Thus, this strategy can be used to monitor the efficacy of gene therapy and allow the *in vivo* visualization and tracing of stem cells after transplantation. VZV-TK is another reporter gene associated with herpesvirus. Compared to the system composed of the HSV-TK reporter gene and the corresponding probe, a radiolabelled bicyclic nucleoside analogue (BCNA) with a reporter probe corresponding to VZV-TK can penetrate the blood-brain barrier and is more suitable for PET imaging of central nervous system tumor therapy (Deroose et al., 2012). Recently, some fluorescent probes with more advantages are currently being developed and tested. For example, Shao et al. reported a quantum-dot-based technique for revealing the procedure of HSV-TK/GCV suicide gene therapy by constructing covalent linkages between near-infrared fluorescent quantum dots (QDs) and the TK gene. This stable QD labeling did not influence either the QDs fluorescence or the biological activity of TK gene (Shao et al., 2014). They also developed a nontoxic folate-modified theranostic liposome (FL/QD-TK), which is composed of an HSV-TK suicide gene covalently coupled with near-infrared fluorescent CdSeTe/ZnS core/shell QDs. *In vivo*, FL/QD-TK exhibited highly specific tumor imaging and strong inhibition of the folate receptor-overexpressed mouse xenografts without systematic toxicity (Shao et al., 2015). This application of semiconductor nanocrystalline will promote the development of the integration of cancer diagnosis and treatment.

### Regulation of Parasitic Infection

Most pathogenic human or animal parasites have cell cycles and pyrimidine nucleotide biosynthetic pathways (*de novo* synthesis and salvage pathways) similar to those of higher eukaryotic cells (Radke and White, 1998; Leija et al., 2016). Consequently, the expression of exogenous active viral TK may influence the pyrimidine biosynthetic pathway in the bodies of these organisms, which likely alters their biological features, and cell cycle arrest can be induced by HSV-TK/GCV in a similar manner as in tumor cells (Wei et al., 1998; Zeng et al., 2014). Radke et al. found that the expression of exogenous active HSV-TK in *Toxoplasma gondii* attenuates tachyzoite virulence in mice (Radke and White, 1999). In their study, compared with the parental RH parasites, *Toxoplasma gondii* isolates expressing a chloramphenicol acetyltransferase-HSV-TK fusion sequence (CAT-HSTK) showed no difference in growth rate, host cell invasion rate, or extracellular viability in cell culture but lost lethality to mice. Normally, the parental RH strain caused 100% mortality within 2 weeks at doses as low as 10 parasites; however, doses of up to 10^6^ isolates containing ≥ five copies of the fusion sequence were not lethal to mice, and mice infected with those isolates showed no overt symptoms of disease and were protected from lethal challenge with the parental RH strain. Thus, the attenuated isolate caused by HSV-TK expressing has the prospect of becoming a live vaccine in clinical trials.

The security of these potential vaccines can be ensured by GCV treatment. Buckner et al. demonstrated that *Trypanosoma cruzi* transfected with the HSV-TK gene was sensitive to the nucleoside analogue GCV, and the *in vitro* growth of mammalian life-stage forms (amastigotes and trypomastigotes) was strongly inhibited by GCV (Buckner et al., 1997). Davoudi et al. confirmed in vivo that progressively growing lesions in mice caused by infection with a recombinant *Leishmania major* strain expressing a modified HSV-1 TK gene stably introduced into the chromosome via gene targeting technology were completely cured by 2 weeks of treatment with the sensitive drug GCV (Davoudi et al., 2005). The same conclusion was reached by Davoudi et al.; mice inoculated with a transgenic strain harboring two suicide genes, namely HSV-TK and Cytosine deaminase (CD), were challenged with wild-type *Leishmania major*, and complete protection was induced in mice treated at day 8 (Davoudi et al., 2014). From another point of view, the expression of the HSV-TK gene in parasites also provides an easy, reliable, and sensitive assay for evaluating HSV susceptibility to nucleoside analogues and for assessing the role of specific viral TK mutations (Bestman-Smith et al., 2001).

## Conclusion and Direction

To date, the transcriptional regulation mechanism of the α-herpesvirus TK gene and the basic structure and function of the TK enzyme have been studied in detail. As a crucial virulence gene in α-herpesvirus and a nonessential gene for viral replication, the TK gene not only is a preferred target gene for constructing live attenuated virus vaccines but also provides a superior site for inserting or expressing foreign genes on viral vectors. For example, Yin et al. inserted a bacterial artificial chromosome (BAC) vector and fluorescent protein screening marker into the PRV-TK gene by homologous recombination to form an infectious clone PRV-BAC strain, which provided a good technical platform for researching a recombinant multivalent vaccine with PRV as a vector (Yin et al., 2010). Not long ago, a technology platform was successfully constructed in poultry α-herpesviruses such as DEV and is expected to provide a foundation for the development of new genetically engineered vaccines for poultry (Wu et al., 2017).

Studies about the functions and applications of the TK gene and the encoded enzymes have long focused on antiviral and antitumor applications. Because α-herpesvirus TKs have a wide range of substrate specificities, nucleoside analogues and their derivatives have been used for the clinical treatment of herpesvirus infection for nearly 40 years, with a remarkable curative effect. However, numerous adverse reactions and drug resistance caused by the long-term use of these drugs negatively affect patients. Recently, the proportion of drug resistance in immunocompromised patients has risen sharply, especially the increase in the risk of drug resistance in AIDS patients and transplant recipients (Burrel et al., 2010; Piret and Boivin, 2011; Frobert et al., 2014). Drug resistance is also virus-mediated and is associated to some extent with the acceleration of natural mutations in the α-herpesvirus TK gene. As these trends continue, the development of safer and more efficient new broad-spectrum antiherpesvirus medications that target different stages of viral replication or different types of infected cells will be advanced (Criscuolo et al., 2018).

In antitumor research, although the HSV-TK/GCV system is currently the most deeply researched and widely used suicide gene system and its feasibility and safety have been approved through various experimental tumor models, progress remains unsatisfactory in actual clinical trials. For instance, the poor targeted expression of HSV-TK in transfected cells has always been difficult to overcome. Researchers believe that the most clinically significant approach to combat this limitation is to develop novel gene vectors or effectively enhance the specific expression of HSV-TK by linking transcriptional regulatory elements such as tumor-specific promoters and enhancers and identifying the appropriate gene vector is also key to guaranteeing high security and efficiency in suicide gene therapy (Qiu et al., 2012; Hossain et al., 2016). Besides, combining suicide genes with classical and traditional therapeutic modalities (radiotherapy and RFH) (Chen and Tang, 2010; Ji et al., 2018), developing fusion gene therapy approaches (CD/TK), and designing therapeutic alliances with multiple gene therapies are worthy of exploration. Notably, immune genes, cytotoxic genes and tumor suppressor genes are often the mainstream choices for multi-gene combination therapy strategies, but combination therapy with silencing genes that inhibit immune function is also a feasible reverse method (Ahn et al., 2012). In short, with the continuous augmentation of cancer research and the successful resolution of the above problems, the role of the HSV-TK suicide gene therapy will become increasingly prominent.

## Author Contributions

All authors listed have made a substantial, direct, and intellectual contribution to the work and read and approved the final manuscript.

### Conflict of Interest Statement

The authors declare that the research was conducted in the absence of any commercial or financial relationships that could be construed as a potential conflict of interest.
